# Adobe Bricks of the Champagne Region (France): Characterization of a Chalky Raw Earth Construction Material

**DOI:** 10.3390/ma17102307

**Published:** 2024-05-13

**Authors:** Guillaume Polidori, Adrien Aras-Gaudry, Fabien Beaumont, Fabien Bogard, Sébastien Murer, Mohammed Lachi, Chadi Maalouf, Tala Moussa, Christophe Bliard, Gilles Fronteau, Erwan Hamard

**Affiliations:** 1 Université de Reims Champagne-Ardenne, Institut de Thermique, Mécanique, Matériaux (ITheMM), F-51100 Reims, France; guillaume.polidori@univ-reims.fr (G.P.); fabien.beaumont@univ-reims.fr (F.B.); fabien.bogard@univ-reims.fr (F.B.); mohammed.lachi@univ-reims.fr (M.L.); chadi.maalouf@univ-reims.fr (C.M.); tala.moussa@univ-reims.fr (T.M.); 2 Université de Reims Champagne-Ardenne, Groupe d’Étude sur les Géomatériaux et ENvironnements Anthropisés (GEGENA), F-51100 Reims, France; adrien.aras@univ-reims.fr (A.A.-G.); gilles.fronteau@univ-reims.fr (G.F.); 3 Université de Reims Champagne-Ardenne, Institut de Chimie Moléculaire de Reims (ICMR), UMR 7312 CNRS, F-51100 Reims, France; christophe.bliard@univ-reims.fr; 4 Université Gustave Eiffel, Matériaux et Structures—Granulats et Procédés d’Élaboration des Matériaux (MAST-GPEM), F-44340 Bouguenais, France; erwan.hamard@univ-eiffel.fr

**Keywords:** adobe bricks, building materials, earth construction, compressive strength, hygrothermal performance

## Abstract

Raw earth bricks made from the soil of the Chalky Champagne region (France) have been used for at least two millennia in construction, a promising heritage in the context of reducing the carbon emissions of buildings. The present experimental study aims to measure the physical, mechanical, thermal, and hydric properties of adobes collected from a local village barn. The results show a high chalk content, estimated at 71%, and a clay content, acting as a binder, of 14%. Despite limited load-bearing capacity, these lightweight adobes are suitable for current single-story constructions, while their hydrothermal properties classify them as excellent moisture regulators for occupants. In association with other bio-sourced materials such as starch–beet pulp bricks, Chalky Champagne adobes yield promising insulating properties, and meet the criteria defined by current energy standards.

## 1. Introduction

Since the 1970s and the first oil crisis, the construction sector has undergone a series of changes that have had an impact on the way in which buildings are built, moving towards a more environmentally friendly approach. But the reality shows that fifty years later, this target is far from being achieved. At a global scale, the emissions of the building and construction sectors are still rising, and represent 37% of global operational energy and process-related CO_2_ emissions, demanding a fundamental shift in this sector to create a credible path to tackle climate change [1]. In France, these changes primarily concern energy consumption for building use through various thermal standards. Since then, improvements have been made to reduce operational energy and embodied carbon emissions. From 1 January 2022, the 2020 French Environmental Regulations [2] have required buildings to reduce their carbon footprint while continuing to improve their energy performance and comfort. One way of achieving this goal is through taking the Life Cycle Assessments (LCAs) of construction materials into account. To achieve these objectives, professionals in the construction sector are seeking to relocate the resources used in building [3]. This is part of a wider trend that seeks to establish ethical values behind the notion of living [4], while reinterpreting ancient know-how and the notion of the vernacular [5]. It is within these various movements that raw earth has seen a resurgence of interest in recent years and is the subject of a growing number of studies in the scientific world. The advantages of this material are many: low environmental impact [6], excellent regulation of indoor hygrothermal comfort [7], regulation of indoor air quality [8], acoustic qualities [9], and recyclability [10]. Moreover, raw earth is considered a waste product by construction sites and therefore constitutes a resource already available. The global waste production in France reached 315 million tons in 2021, with 213 million tons (68%) produced by the building sector, half of which being earth [11]. The very presence of earthen heritage in a given area demonstrates its ability to last over time and to meet the resource needs of local construction [12,13]. This heritage is therefore a source of inspiration for future construction [14,15]. The soil used in construction can vary considerably depending on its location and is dependent on geology. Studying this allows us to highlight specific skills and also to gain a better understanding of the characteristics associated with a given soil in a given geographical area. Different types of barriers slow the development of earth construction [16]. Lack of knowledge and cultural beliefs are one of these barriers [17]. Developing this knowledge from earthen heritage local architecture could help tackle some of these barriers. Adobe construction in the Champagne region has been infrequently studied, often alongside stone heritage [18] or for earlier periods [19]. These raw earth bricks, known locally as “carreaux de terre”, have been frequently used in the area for at least two thousand years [20,21] and cover a vast area, and are mainly characterized by limestone elements corresponding to the soil and sub-soil of the Chalky Champagne region [22]. Our study aims to reveal the physical and mechanical characteristics of bricks taken from the heritage of the Chalky Champagne region to demonstrate both that it is a quality material for existing buildings, and that it has major advantages for new local construction under the new French environmental regulations as an ecological building material with a high comfort value.

## 2. Materials and Methods

### 2.1. Survey Location Specificity and Origin of Samples

**Figure 1:**
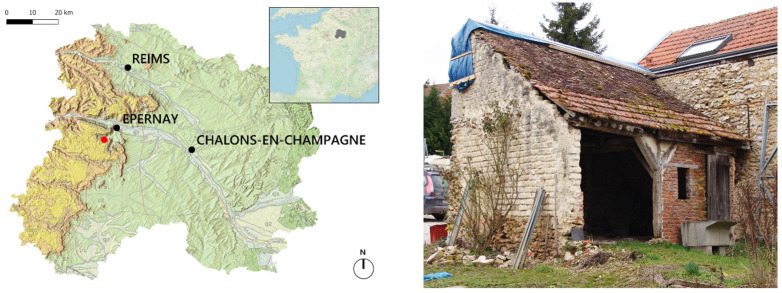
Sampling site at red dot (**left**); building before demolition (**right**).

The research presented in this paper is based on adobe bricks sourced from the demolition of a small old barn in a village called Brugny-Vaudancourt, Marne, North-Eastern France (see Figure 1). The village is located at the bottom of the Île-de-France cuesta slope and at the start of the Chalky Champagne plain, characterized by its Upper Cretaceous chalk substratum. The dominant soil types are calcosols and rendosols, corresponding to soils developed from limestone, frequently clayey and rich in carbonates.

### 2.2. General Characteristics of Adobes

**Figure 2:**
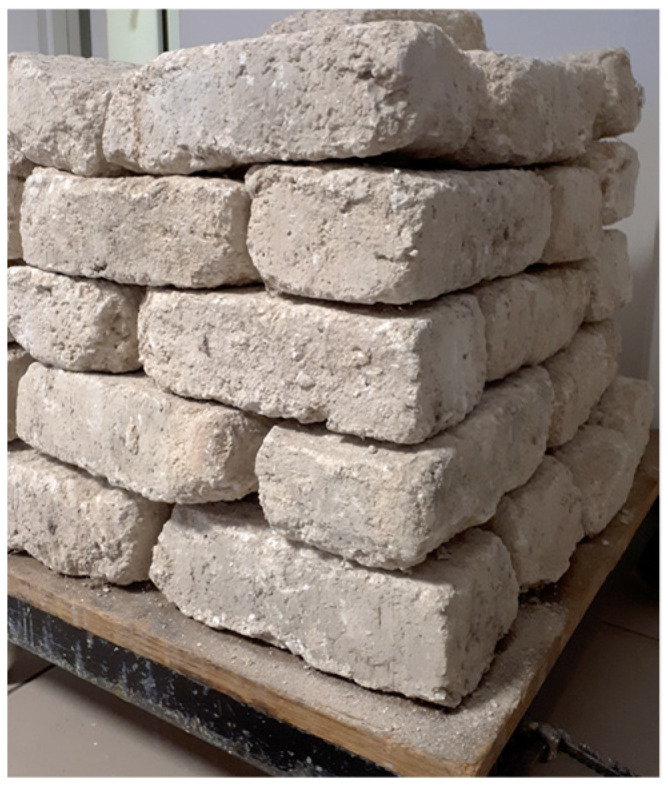
Adobes from the Champagne region (France).

Approximately fifty adobes were obtained for analysis (see Figure 2). Their light ochre hue indicates their origin from cretaceous chalky soil. Following the local literature [23], they appear to have been manually shaped on-site using rectangular molds of varying yet closely similar sizes. Measurements conducted on a representative sample revealed the following dimensions (expressed as mean values ± standard deviations): width (W) = 139.2 mm (±8.4 mm); height (h) = 85.1 mm (±6.2 mm); and length (L) = 264.5 mm (±12.6 mm). Based on the mass of M = 4556.2 g (±418.2 g), the average apparent density $\rho_{app}$ = 1464 kg/m^3^. Within construction and masonry, the adobe bricks tested can be classified as lightweight, which may limit their load-bearing capacities. However, this characteristic also suggests quite interesting thermal properties. The latter is supported by the average absolute density measurement ($\rho_{abs}$ = 2240 kg/m^3^), which indicates a porosity of 34.6%. All tests were conducted on adobe bricks with a measured % moisture content of 2% (dry density 1435 kg/m^3^). Additionally, the pH value was determined to be 8.5, while the soluble organic matter content was 0.5% by mass (evaporation of supernatant obtained after wet sieving and centrifugation).

### 2.3. Particle Size Distribution

After a preliminary visual examination of fractured bricks and sliced pieces cut with a circular saw, granulometric analysis was conducted on five randomly selected samples to gain further insight into the adobe composition. Particle distribution analysis for sizes between 63 µm and 20 mm was achieved by wet sieving, while sedimentometry analysis was used for smaller sizes in adherence with the standard NF EN ISO 17892-4 [24].

### 2.4. Methylene Blue Value (MBV_1_)

The Methylene Blue Value (MBV_1_, distinct from the moisture buffer value to be discussed later, denoted as MBV_2_) represents a commonly utilized technique for detecting the presence of clay minerals within soils. Our investigation enables the characterization of clay content in the soil from which the examined raw clay bricks are derived, as well as their susceptibility to water, a critical parameter in construction applications. While various methodological variations exist for conducting the methylene blue test [25], we adhered to the specifications outlined in the French standard NF P 94-068 [26]. This procedure involves incrementally introducing methylene blue into a soil suspension while agitating it. Periodically, a drop of the suspension is extracted and deposited onto chromatographic paper. The completion of methylene blue adsorption onto clay particles is indicated by the development of a bluish halo around the initial blue spot formed after drop deposition, signifying the presence of excess methylene blue in the halo. MBV measurements, expressed in grams of blue per 100 g of soil, were conducted on approximately a dozen randomly selected samples from different bricks.

### 2.5. Compression Tests

The compression tests were conducted using a Zwick Roell Z050 (Ulm, Germany) testing machine, which was fitted with a 50 kN load cell. These tests took place under controlled indoor conditions, with a room temperature of 23 °C and 50% relative humidity. The compression rate adhered to the standard specifications of NF XP P 13-901 [27], set at 8 mm/min, which caused the fracture of the specimens after a duration of between 1 and 2 min.

For statistical robustness, compressive strength analysis typically involves averaging results from 5 to 10 samples [28]. In this study, seven adobe specimens were subjected to testing. Various standards about earthen construction emphasize the mechanical testing of bricks; however, there is limited consensus regarding specimen geometry [29]. To maintain consistency with the in situ load conditions of adobes in walls, the thickness of the specimens remained unaltered. Only the compressed surface area of 10 × 10 cm^2^ necessitated manual trimming of the adobe. To ensure optimal contact between the specimen faces and compression platens and to minimize the effects of any extraneous loading, the faces were manually smoothed using sandpaper.

### 2.6. Three-Point Flexural Tests

The Zwick Roell Z050 testing machine, equipped with a 50 kN load cell, was also used for 3-point bend tests. To avoid edge effects, the ratio of support-to-edge distance/adobe length was set as being greater than 1/8. The support span was $L_{s}$ = 165 mm. Assuming pure bending and linear elastic material behavior, the three-point bending test enables the evaluation of the flexural tensile strength $\sigma_{f}$, commonly referred to as “modulus of rupture”, and the flexural strain $\varepsilon_{f}$. Flexural tests were conducted on 7 specimens.

### 2.7. Thermal Analysis

Thermal properties were assessed using the thermal characteristics analyzer device ISOMET 2114 (Applied Precision, Ltd., Bratislava, Slovakia) based on heat flux pulses applied at the material surface. Five specimens were analyzed to determine the thermal conductivity, specific heat, and diffusivity of the adobes.

### 2.8. Moisture Buffer Value (MBV_2_)

The moisture-buffering value assesses a material’s capacity to moderate fluctuations in relative humidity within an enclosed space. Essentially, the MBV_2_ value reflects whether a material effectively regulates moisture, impacting user comfort and other factors. Measurement follows the protocol outlined in the Nordtest Project [30], allowing the classification of moisture-buffering values from negligible to excellent. Initially, samples were stabilized at 23 °C and 50% relative humidity (RH) for 14 days, then subjected to daily RH cycles: 8 h at high RH (75%) followed by 16 h at low RH (33%) within a climatic chamber (Binder MKF 720, Tuttlingen, Germany). Cycles continued until the difference in measured mass variations across the last three cycles was below 5%. The MBV_2_ value was determined using the following equation:

(1) $${MBV}_{2}=\frac{∆m}{A\;({RH}_{sup}-{RH}_{inf})}$$

where MBV_2_ represents the moisture buffer value in units of grams per square meter per percent relative humidity (g/(m^2^.%RH)), Δm denotes the mass variation during absorption or desorption in grams, A denotes the sample surface area in contact with air in square meters (m^2^), and ${RH}_{sup}$ and ${RH}_{inf}$ denote the upper (75%) and lower (33%) relative humidity levels, respectively.

Four parallelepiped samples measuring 10 × 10 × 4 cm^3^ were utilized as test specimens and sealed on the side and bottom surfaces with waterproof adhesive tape.

## 3. Results

### 3.1. Particle Size Distribution

**Figure 3:**
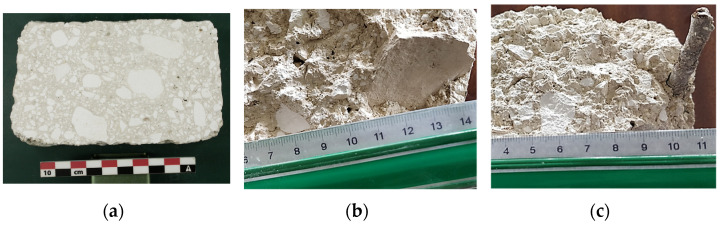
(**a**) Details of adobe constitution; (**b**) pottery piece; (**c**) tree branch.

A preliminary visual examination of the fractured or cut bricks indicates a heterogeneous grain size distribution, characterized by the presence of numerous chalk gravels and pebbles (Figure 3a). The adobe composition is mineral, devoid of any plant fibers. However, fragments of pottery (Figure 3b) or tree branches (Figure 3c) were incidentally found within the adobe structure, suggesting that the material was sourced directly from on-site soil.

**Figure 4:**
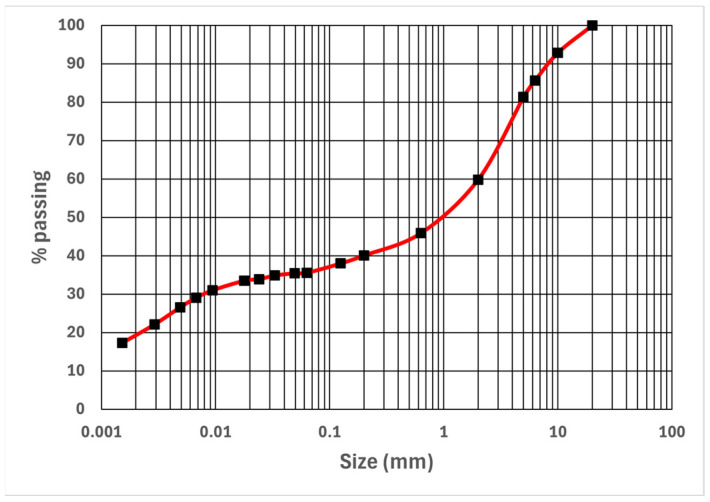
Particle Size Distribution of the earth bricks.

Although minimal, the presence of these constituents suggests a potential dispersion in the reproducibility of these brick properties, potentially resulting in localized mechanical weaknesses or diminished particle cohesion. Figure 4 illustrates the mean granulometric curve. The distinctive nature of this adobe type stems from the highly chalky soil from which it originates. The elevated material levels at low % passing can be attributed to the abundant presence of chalk micrograins (such as coccoliths with sizes below 12 µm) and fine loam [31,32], alongside clay. The Particle Size Distribution reveals that particles less than 2 µm are estimated at around 20% by mass. Nevertheless, chalk-based earth adobes are particular in that not only fine clay particles but also chalk micro- and nanoparticles and small coccoliths are present in this range. Applying a decarbonization process to the smallest particles yields an estimation of clay content close to 14%.

### 3.2. Methylene Blue Value (MBV_1_)

**Figure 5:**
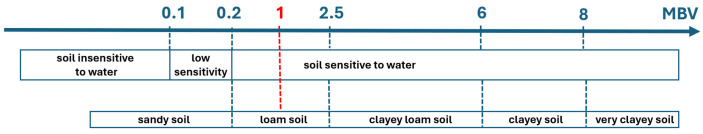
Schematic soil characterization and classification according to water sensitivity, from [33].

The measured MBV1 value was 0.97 ± 0.16, which can be approximated as unity. This value is depicted in the diagram presented in Figure 5, which schematically outlines the soil characterization and classification ranges based on water sensitivity.

The soil extracted for the earthen bricks can be categorized as loamy and water-sensitive, following the guidelines outlined in the technical manual for road earthworks published by the French Ministry of Transport, known as the “Guide des Terrassements Routiers” [33]. As per the Particle Size Distribution studied (Figure 4), the percentage passing through the 80 μm sieve is approximately 37%. By combining this value with the Methylene Blue Value (MBV), as proposed by Rojat et al. [34], the earth material can be classified. According to the GTR A1 classification, the material is fine soil with low plasticity, exhibiting the ability to undergo rapid changes in consistency with slight fluctuations in water content. This classification denotes fine soils with low plasticity that can exhibit abrupt changes in consistency due to minor variations in water content. The response time to changes in the moisture and climatic conditions is relatively short.

Decarbonization was conducted using a lab-built single-unit Scheibler apparatus following the NF EN ISO 10693:1995 [35] standard methods, yielding a carbonate content of 71% in the samples. This is in good agreement with the clay content obtained by sedimentometry and was found to be around 14%. The overall clay content, serving as the binding agent in the material, must strike a balance between providing adequate mechanical strength and preventing cracking and shrinkage. The literature suggests that clay content ranging from 5% to 29% in adobe is considered acceptable [28,36,37].

### 3.3. Compression Tests

**Figure 6:**
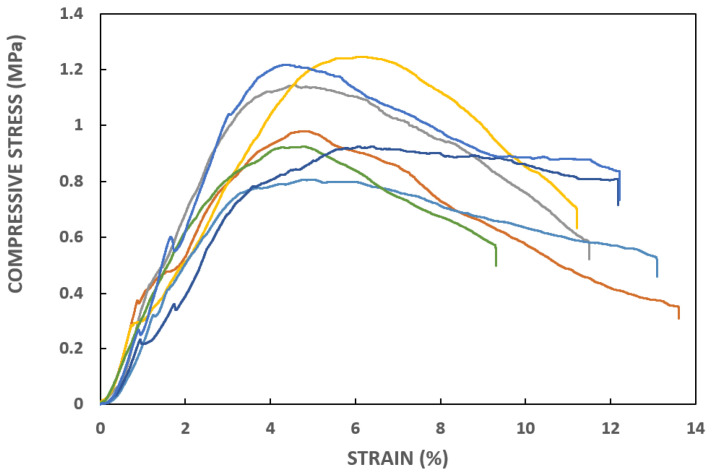
Stress–strain experimental curves from compression tests.

**Figure 7:**
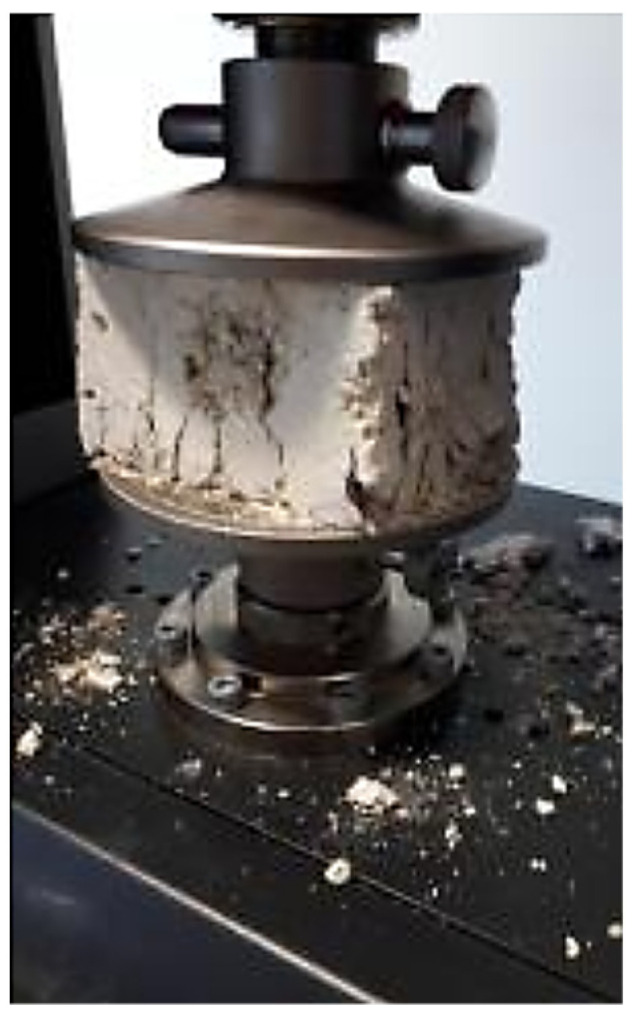
Presence of vertical cracks.

Uniaxial compression tests were conducted on all seven specimens, resulting in the stress–strain curves depicted in Figure 6, highlighting notable variability in compression behavior. Such an observation may be attributable to varying clay content among the bricks, but also to differences in compaction during the manufacturing process or weathering. In all tested samples, failure occurred along sub-vertical planes intersecting the top and bottom surfaces of the specimens (Figure 7). Table 1 summarizes the mechanical properties observed, including the peak compressive strength f and the axial strain at peak compressive strength $\varepsilon_{u}$.

Compressive strength is one of the pivotal mechanical parameters influencing material selection in building construction. In this investigation, the mean peak stress was determined to be f = 1.03 MPa, aligning with the values reported in prior studies [38,39,40,41]. This finding is consistent with certain literature recommendations; for instance, the standard XP P13-901:2022 [27] stipulates that the compressive strength of dry earth bricks typically falls within the range of 0.6 to 2.0 MPa. Notably, Doat et al. [42] proposed a compressive strength of 2 kg/cm^2^ for single-story construction. In our investigation, an average compressive strength of 10.3 kg/cm^2^ was observed, surpassing this recommendation by fivefold.

**Figure 8:**
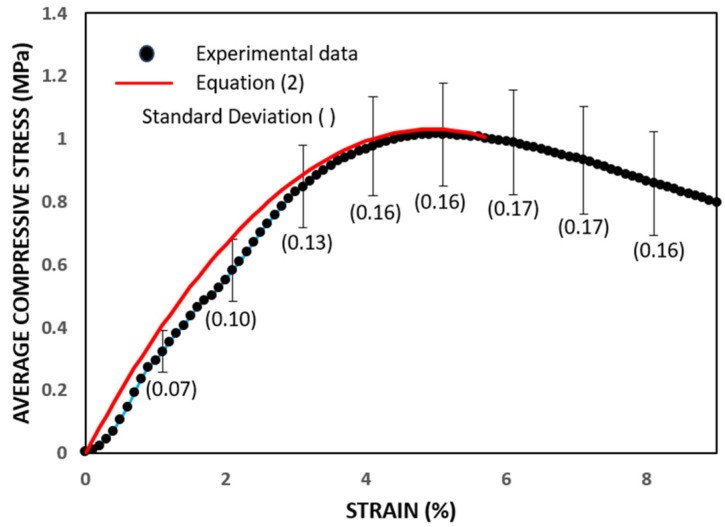
Comparison between the average experimental curve and the proposed model.

To determine the Young’s modulus (initial tangent modulus), the mean stress–strain curve is illustrated in Figure 8. It is evident that the experimental curve closely conforms to the theoretical model proposed by Adorni et al. [29], which can be expressed as:

(2) $$\sigma =f\left[2\frac{\varepsilon }{\varepsilon_{u}}-{\left(\frac{\varepsilon }{\varepsilon_{u}}\right)}^{2}\right]$$

The observation of quasi-linear behavior up to 0.8 f enables us to infer an estimate of Young’s modulus, approximately E = 32 MPa.

The initial tangent modulus values seem widely dispersed in the literature, stemming from the diversity of soil composition within adobes and the lack of standardization in the manufacturing process. Nonetheless, the value of 32 MPa aligns closely with findings reported by Illampas et al. [43] and Fratini et al. [44].

### 3.4. Three-Point Flexural Tests

**Figure 9:**
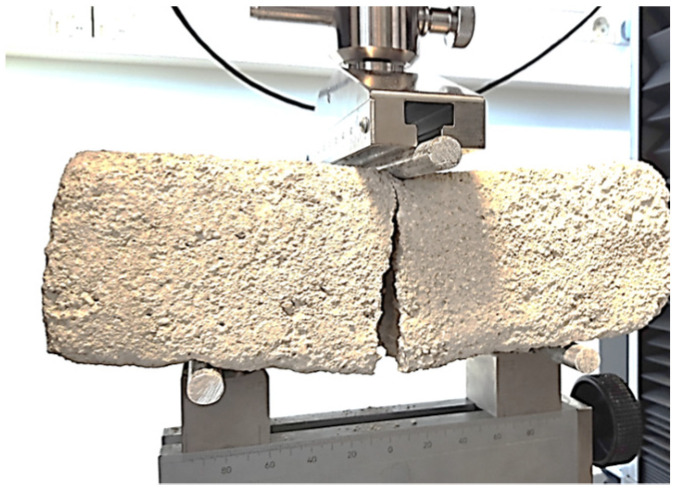
Experimental setup for 3-point bending.

The outcomes of the flexural tests shown in Figure 9 are consolidated in Table 2.

In our experiments, due to the brick dimensions, the specimens exhibited a span-to-depth ratio of L/h = 1.8–2, significantly lower than the theoretical value necessary for pure bending (L/h > 4). However, none of the samples exhibited an arched crack, suggesting that the assumption of the linear elastic behavior of the material is substantiated by the linear trend observed in the force–displacement curves. Consequently, the flexural stress can be approximated as follows:

(3) $$\sigma_{f}=\left[\frac{3\;F\;L}{2\;b\;h2}\right]$$

**Figure 10:**
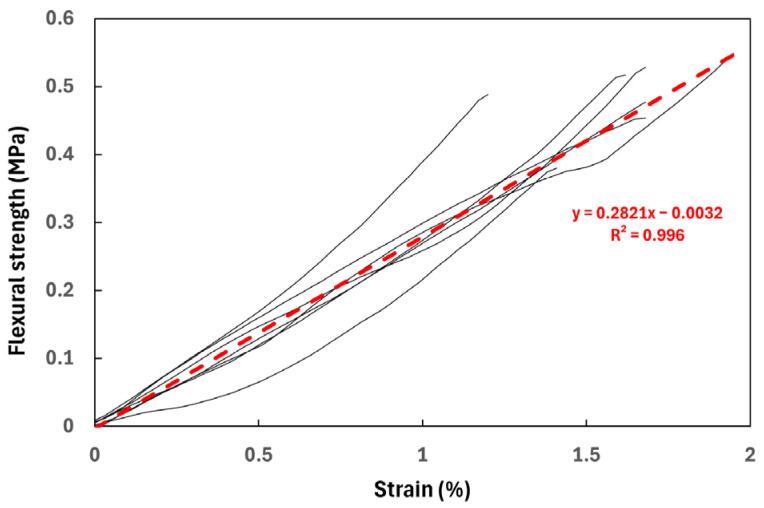
Stress–strain curves for 3-point bending tests (vertical fracture drop not displayed).

The stress–strain curves derived are depicted in Figure 10, revealing notably reduced dispersion in comparison to the compression curves. Linear interpolation yields excellent outcomes, with a correlation coefficient R^2^ = 0.996 and a calculated flexural modulus of 28.2 MPa, closely resembling Young’s modulus obtained in the compression test. Relative to the mean values of peak stress (1.03 MPa) and flexural stress (0.48 MPa), corresponding standard deviations (i.e., dispersion of the experimental results), namely 0.17 and 0.06 MPa, highlight comparable orders of magnitude.

Comparisons of the primary flexural values can be made with the established literature standards. In the case of flexural strength (0.48 MPa in the present study), the standard NZS 4298:2020 [45] suggests a minimum of 0.25 MPa, while both PCDS [46] and RLD [47] propose a minimum of 0.34 MPa.

### 3.5. Thermal Analysis

Table 3 presents the mean values and standard deviations, and provides the thermal properties of common load-bearing materials such as oak wood, solid brick, and conventional concrete for comparison purposes.

A material is typically deemed an excellent insulator if its thermal conductivity λ is below 0.03 W/(m.K) according to the standard DIN 4108 [48]. The thermal conductivity of adobe was 0.67, indicating satisfactory insulating properties, better than that of commonly used geo-sourced load-bearing materials such as fired brick or concrete. This value aligns with the typical thermal conductivity range of adobes, 0.5–1.2 W/(m.K), as suggested by Rempel and Rempel [49]. This might lead us to think that to achieve a given thermal resistance, adobe would have to be 1.6 times thinner than conventional brick and 3.1 times thinner than ordinary concrete, thus underlining its eco-friendly nature by minimizing the volume of construction materials required. However, this does fail to consider its significantly lower compressive strength, and thermal resistance cannot be disconnected from mechanical requirements, so the right balance needs to be struck.

**Table 3:** Thermal properties of adobe (highlighted in grey) and other conventional load-bearing materials [50].

	Thermal Conductivity λ (W/(m.K))	$\mathrm{Specific}\;\mathrm{Heat}\;\mathrm{Capacity}\;c_{p}$ (kJ/(kg.K))	Diffusivity a (10^−6^ m^2^/s)
Adobe (present study)	0.669 (0.033)	1.109 (0.004)	0.414 (0.019)
Wood oak	0.17	1.6	0.15
Plain brick	1.10	0.9	0.61
Plain concrete	2.1	1.0	0.83

In the realm of building construction, thermal diffusivity manifests itself through temperature fluctuations in the outdoor environment and represents a material’s capacity to store thermal energy. Essentially, it quantifies thermal inertia, a pivotal factor in the thermal comfort of a building regardless of the season. A lower thermal diffusivity value implies a longer duration for the heat front to penetrate the material’s thickness, thereby enhancing thermal comfort. The thermal diffusivity of adobe, as indicated in Table 3, suggests that a structure made of adobe would dampen outdoor conditions better than concrete but less effectively than wood.

The specific heat capacity $c_{p}$ [J/(kg.K)] delineates the amount of energy needed to raise the temperature of 1 kg of adobe by 1 K (=1 °C). Accurate determination of heat capacity is crucial as it serves as a significant parameter for predicting heat transfer capability. A higher mass thermal capacity of a building material translates to a greater heat requirement for temperature elevation. The obtained average value of 1.0 kJ/(kg.K) is in very good agreement with the one reported by Yan et al. [51] of 0.902 kJ/(kg.K) and by Eben Saleh [52] of 1.0 kJ/(kg.K) for rammed earth and adobe, respectively. Relative to other load-bearing materials, adobe exhibits a specific heat about 11–23% higher than mineral-based materials but 31% lower than organic ones.

### 3.6. Moisture Buffer Value (MBV_2_)

**Figure 11:**
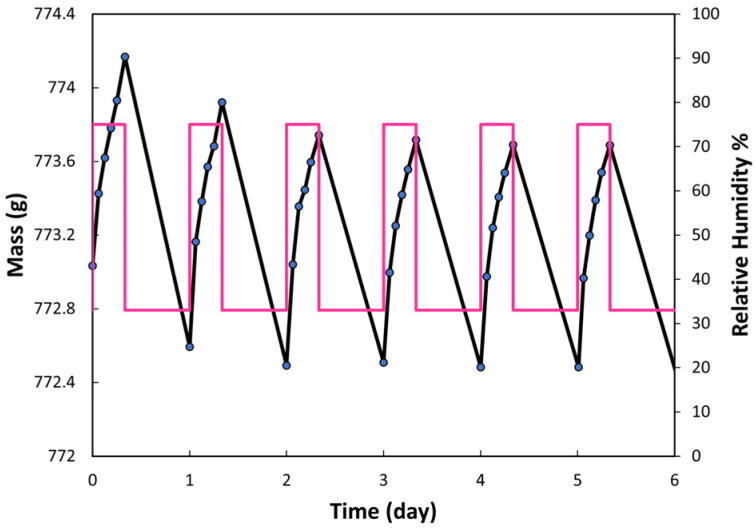
Average moisture absorption–desorption cycles of adobes (red line denotes the daily RH cycles, and black line the weight of the selected sample).

The samples underwent weighing five times during the absorption phase and twice during the desorption phase. Variations in the weight of the samples during absorption/desorption dynamics are depicted in Figure 11. Mass changes stabilized for the last three cycles, facilitating the calculation of the MBV_2_ value using Equation (1). The calculated MBV_2_ value is 2.54 (0.40).

**Figure 12:**
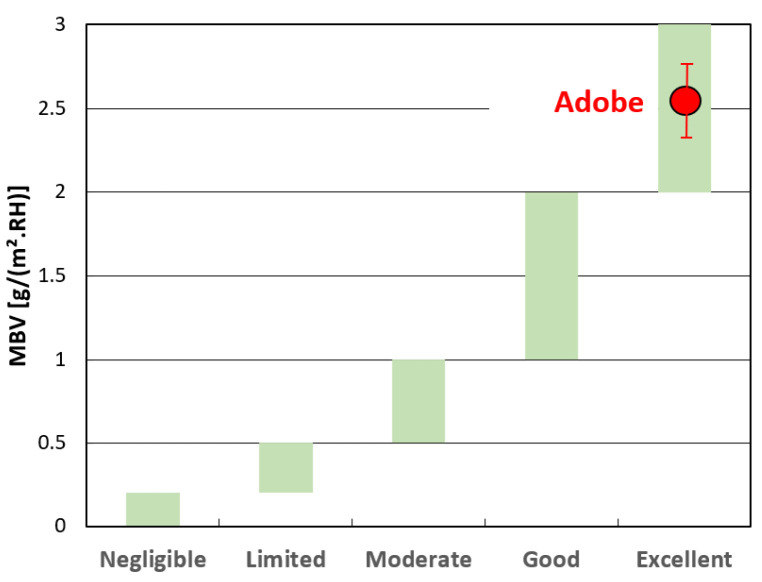
Nordtest project classification [30].

Materials exhibiting a high MBV_2_ value possess the capability to mitigate indoor humidity variations, thereby enhancing air quality, reducing microbial growth phenomena, and potentially lowering respiratory health risks. The Nordtest project [30] has established a classification of moisture buffer values ranging from negligible to excellent. Figure 12 illustrates this classification, with the current measured value for adobe positioned accordingly.

Following the Nordtest protocol, the examined adobe samples were categorized as excellent moisture regulators, indicative of values surpassing 2 g/(m^2^.%RH). For example, for other load-bearing structural materials such as masonry bricks and concrete, the MBV values can be estimated at 0.48 and 0.37, respectively, classifying them as limited-effect moisture regulators [53].

### 3.7. Simulation of Adobe Integration within a Practical Eco-Friendly Vertical Wall

**Figure 13:**
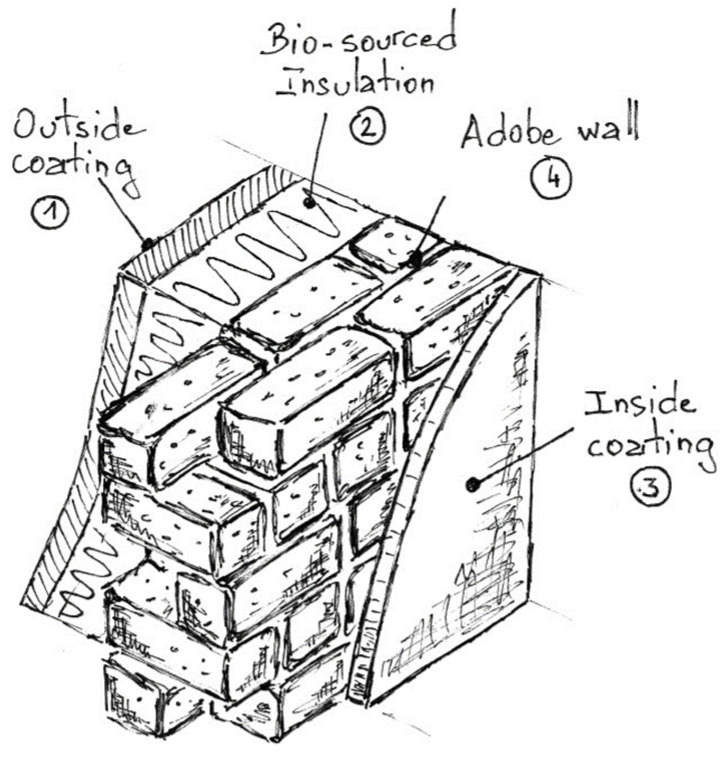
Example of wall configuration.

An examination of the thermal characteristics of adobe naturally prompts consideration of its application in residential walls. Let us envision a feasible and environmentally conscious composite wall configuration, comprising load-bearing adobe as the structural core, bio-sourced insulation nestled within, and interior and exterior finishes crafted from earth and hemp concrete with hydraulic lime as the binder, respectively, to ensure effective weatherproofing. Insulation would be from the outside, as adobe improves summer comfort by preventing overheating in summer and regulating humidity. Naturally, such a structure would be single-story and designed to accommodate minimal permanent (ceiling and roof) and operational loads. This composite wall assembly could adopt a design and composition akin to those depicted in Figure 13 and detailed in Table 4.

It could be postulated that the mortar joints between the adobe bricks share the same composition as the adobe material itself, as does the interior plaster. Similarly, the insulation bricks, made entirely from bio-sourced materials such as beet pulp and starch (both originating from the same French region as the adobe), could be vertically arranged in the walls, with joints filled using the same bio-sourced material [54]. The exterior cladding may consist of hemp concrete, a locally sourced product currently under experimentation [55]. To ensure perfect adhesion and capillary continuity through the wall, no air layer is considered. Under this configuration, which is pragmatically feasible in construction terms, the pertinent question arises regarding the required thickness of the load-bearing structure (i.e., the adobe wall) to comply with prevailing French ecological transition standards. The new energy and environmental regulations, RE2020, for newly constructed buildings, as stipulated by the French public authorities, mandate that the thermal resistance (R-value) of a wall falls within the range of 2.2 ≤ R [m^2^K/W] ≤ 2.9. This criterion aims to reduce energy consumption during both winter (heating) and summer (air conditioning) seasons. While higher R-values indicate superior thermal insulation, they must be balanced with wall thickness considerations.

**Table 4:** Denomination of possible used wall materials. (*) from [55]; (**) from [54].

	Designation	Thickness e (m)	Thermal Conductivity λ (W/m.K)
①	Hemp concrete outside coating	0.05	0.095 (*)
②	Fully bio-sourced insulation	0.16	0.09 (**)
③	Earth-based inside coating	0.015	0.67
④	Adobe (present study)	*TBD*, $e_{a}$	0.67

Take, for instance, the maximum value from the previous context, specifically R = 2.9 m^2^K/W. What thickness of the adobe wall would be necessary to achieve this threshold? Is the thickness mentioned above feasible in practice? The thickness of the adobe within the composite wall is determined by the following equation, derived from the expression of the thermal resistance of the composite wall:

(4) $$e_{a}\geq \lambda_{a}\left[R-\frac{1}{h_{i}}-\frac{1}{h_{e}}-\sum_{i=1}^{3}\frac{e_{i}}{\lambda_{i}}\right]$$

where $1/h_{i}$ and $1/h_{e}$ represent the internal and external surface resistances, respectively, denoting the heat transfer from or to the surface of a building component from its surrounding environment. These values are considered constant and are specified as 0.13 m^2^K/W and 0.04 m^2^K/W, respectively [56].

**Figure 14:**
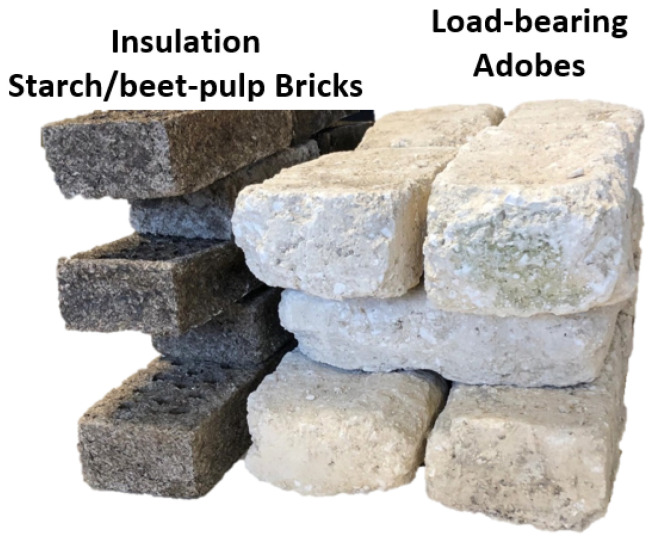
Laboratory reconstruction of load-bearing adobe wall and insulating bio-sourced wall (external and internal coatings, as well as joint mortars not depicted).

A thickness value of adobe $e_{a}$ greater than or equal to 27 cm is determined, which approximately corresponds to doubling the thickness of a row of adobe bricks studied. This aligns with contemporary construction methods for load-bearing structures. Indeed, utilizing earth-based materials, best practices recommend constructing load-bearing walls with a thickness of 30 cm. This involves alternating a row of bricks laid lengthwise (i.e., along the length of the brick, perpendicular to the wall face) with a row of bricks laid widthwise (in this case, two bricks side by side are required to achieve the desired thickness), as illustrated in Figure 14.

## 4. Conclusions and Perspectives

This study focused on traditional manufactured raw earth bricks from the Chalky Champagne region. Numerous chalk pebbles are present, confirmed by a high carbonate content representative of the local geology. These bricks are considered to be fine soils with low plasticity according to the French technical manual for road earthworks “Guide des Terrassements Routiers” [33], and are therefore subject to abrupt changes due to minor variations in water content, confirming that those materials need protection from the weathering and capillary rise, which is not always the case according to field observations [22]. Mechanical tests (compression and three-point flexure) and thermal analysis of the bricks are in line with the literature on adobe bricks which do not contain carbonate. For a single-story construction, the mechanical behavior exceeds the recommendations for compressive strength by a factor of 5, and those for flexural strength by a factor of 1.4 to 1.9. This confirms the presence of an adobe heritage, showing that local earth resources are sufficient for building. Thermal analysis shows that this earth material is good for dampening outdoor conditions, ensuring the comfort of inhabitants during the summer heat. According to the Nordtest project [30], adobe samples are considered excellent moisture regulators, a feature that could be taken into account while designing new projects or renovating earth heritage in the region, to improve air quality and mitigate indoor humidity without energy dependency. A simulation of adobe integration within a practical eco-friendly vertical wall composed of biodegradable materials from the same region has opened good perspectives for complying with French energy and environmental regulations. This first study on the physical and mechanical characteristics of these bricks from the Chalky Champagne heritage demonstrates that the local earth resources can lead to major advantages for new local construction in line with the shift needed in the construction sector [1], whereas in the three departments concerned with this adobe construction, around 1 million tons of earth were produced and considered as waste by the construction sector in 2021 [57]. Nevertheless, chalky soils are not the only resource used to produce adobe in this heritage area [22], and further studies should be carried out on different specimens from other geological bedrocks in order to be able to characterize the greater diversity of local earth resources more precisely. Finally, earth can be used as a binder for natural and local insulation, as studied in the CobBauge project [58,59]. As a small quantity of raw earth is needed to produce light earth, an investigation on the possibility of producing an insulating material based on local chalky earth could demonstrate new possibility for renovating local heritage and designing low-carbon buildings with a high comfort quality.

## Figures and Tables

**Table 1:** Mechanical properties of adobe bricks in compression.

Sample	1	2	3	4	5	6	7	Average (SD)
Peak stress, f (MPa)	0.98	1.14	1.25	0.79	0.92	0.92	1.21	1.03 (0.17)
$\mathrm{Peak}\;\mathrm{strain},\;\varepsilon_{u}$ (%)	4.85	4.57	6.08	4.21	4.27	6.40	4.25	4.95 (0.91)

**Table 2:** Geometric and mechanical properties of adobe bricks in flexure.

Sample	1	2	3	4	5	6	7	Average (SD)
Width b (mm)	145	138	145	129	138	134	152	140.1 (7.7)
Thickness h (mm)	80	84	86	81	81	82	83	82.4 (2.1)
F (N)	1832	1786	2242	1808	1390	1990	1749	1828 (258)
Flexural stress $\sigma_{f}$ (MPa)	0.49	0.45	0.52	0.53	0.38	0.55	0.41	0.48 (0.06)
Flexural strain $\varepsilon_{f}$ (%)	1.19	1.66	1.61	1.67	1.40	1.96	1.68	1.60 (0.24)

## Data Availability

Data are contained within the article.
